# Human preleukaemia: do we have a model?

**DOI:** 10.1038/bjc.1987.1

**Published:** 1987-01

**Authors:** A. Jacobs


Br. J. Cancer (1987), 55, 1–5 © The Macmillan Press Ltd., 1987

REVIEW

### Human preleukaemia: Do we have a model?

A. Jacobs

*Department of Haematology, University of Wales College of Medicine, Heath Park, Cardiff, UK.*

Our perception of preleukaemia as a clinical syndrome has changed during the last 5–10 years. Block *et al.* (1953) observed that it was sometimes difficult to determine the exact time at which a particular patient with refractory anaemia might be classified as leukaemic and that in any case the diagnosis of preleukaemia could only be substantiated finally by the terminal appearance of overt clinical leukaemia. More recently, the preleukaemic states have been defined more precisely and the French–American–British (FAB) group have proposed that all these conditions are grouped together as the myelodysplastic syndromes (MDS) (Bennett *et al.*, 1982). It is now generally accepted that this represents clonal abnormalities of haemopoietic stem cells characterised by a variety of phenotypic manifestations and with a high risk of eventual leukaemic transformation (Greenberg, 1983; Jacobs, 1985). The clinical syndromes of refractory anaemia, with or without sideroblasts, and with high or low numbers of blast cells in the bone marrow, have been fully described in recent reviews (Francis & Hoffbrand, 1985; Jacobs, 1985).

Increasing clinical awareness has resulted in the diagnosis of preleukaemic states at an earlier stage in their development but we still have no clear conception of how the abnormality arises or progresses to its eventual overtly malignant termination. In attempting to describe a model for this process we need to take account of the available clinical data in this condition, current hypotheses regarding the mechanism of malignant transformation and the probable identity of the target cell in the haemopoietic system.

### Clinical data

Most patients with MDS are anaemic and a wide range of morphological, immunological and functional red cell abnormalities have been described (Jacobs, 1985). There may be a variety of metabolic abnormalities (Valentine *et al.*, 1973; Cetto *et al.*, 1982), the reappearance of haemoglobin F (Richard *et al.*, 1979), acquired haemoglobin H (Boehme *et al.*, 1978) and changes in membrane antigens (Levine *et al.*, 1984). These changes are associated with gross dyserythropoietic appearances in the bone marrow with both nuclear and cytoplasmic abnormalities. There may be erythroid hyperplasia, and in such cases sideroblastic granules indicating mitochondrial iron deposition can usually be found in a varying proportion of erythroblasts.

The peripheral blood granulocyte count is usually normal or low and there may be a raised monocyte count. Granulocytes commonly show reduced nuclear segmentation and reduced or absent granules. Cytochemical abnormalities include reduced myeloperoxidase (Schofield *et al.*, 1983) and inappropriately increased α-naphthyl acetate esterase activity (Scott *et al.*, 1983). Abnormalities of both myeloid and macrophage lineage-specific surface markers are common (Clark *et al.*, 1985). Neutrophil function is abnormal in about half of all cases (Boogaerts *et al.*, 1983).

Thrombocytopenia is common and the platelets produced may be abnormal in both morphology (Maldonado & Pierre, 1975) and function (Lintula *et al.*, 1981). Micromegakaryocytes with poor granule formation are common in the bone marrow (Bennett *et al.*, 1982). Occasional patients with sideroblastic anaemia have been reported with an abnormally high platelet count (May *et al.*, 1985; Carroll *et al.*, 1986). Amongst 54 sideroblastic patients seen in Cardiff, 5 had a pathologically high platelet count.

Bone marrow morphology is virtually always abnormal in patients with MDS. Although the combination of hypercellularity with a peripheral blood cytopenia is commonly seen, the marrow may be hypoplastic (Fohlmeister *et al.*, 1985*a*; Frisch & Bartl, 1986). Progression is marked by a gradual increase in the proportion of blast cells. In the FAB terminology, less than 5% blast cells is designated refractory anaemia (RA), 5–20% blast cells refractory anaemia with excess blasts (RAEB) and 20–30% blasts RAEB in transformation. The one year survival following diagnosis in our patients is: primary acquired sideroblastic anaemia (PASA) 95%, RA 64% and RAEB 35%. Most of these patients eventually die from either haemorrhage or sepsis following suppression of normal haemopoiesis and functional inadequacy of the abnormal haemopoietic clone. The classic picture of acute myeloblastic leukaemia only emerges in 17–40% of patients (Foucar *et al.*, 1985; Todd & Pierre, 1986; Coiffier *et al.*, 1983; Greenberg, 1983).

### *Abnormal proliferation of bone marrow cells*

Wickramasinghe (1975) showed that an increased number of polychromatic erythroblasts from patients with PASA were in $G_2$ and a reduced number in S phase, compared to normal, a picture similar to that in vitamin $B_{12}$ deficient marrow. Many cells are arrested in midcycle or in $G_2$ and this state is associated with defective protein synthesis and cell death which is expressed in functional terms as ineffective erythropoiesis. Mitrou and Fisher (1979) made similar observations in six patients with non-sideroblastic refractory anaemia. A parallel study of proliferating myeloid cells showed similar abnormalities in the group as a whole, but within the same marrow specimen it was possible for severe myeloid abnormalities to be accompanied by normal erythropoiesis. A low labelling index for nucleated red cells and myeloid cells in patients with myelodysplastic syndrome has been confirmed by several workers (Hast & Reizenstein, 1977; Seigneurin & Hollard, 1981; Karsdorf *et al.*, 1983). Those patients with the lowest labelling index, indicating the greatest impairment of DNA synthesis, have the poorest prognosis and the highest probability of leukaemic change.

Flow cytometric measurements of DNA in whole marrow confirms that those myelodysplastic patients with the highest proportion of cells in S and $G_2$ phases of the cell cycle have the best prognosis and those with an increased proportion of cells in $G_0/G_1$ have the greatest risk of leukaemic change (Montecucco *et al.*, 1983; Peters *et al.*, 1986).

The evaluation of erythroid production using a mathematical model for the interpretation of ferrokinetic data showed that ineffective erythropoiesis was the major

Received 22 September 1986.

factor causing anaemia in patients with PASA (Barosi *et al.*, 1978). In an extension of this work, quantitative data on total marrow iron turnover, ineffective erythropoiesis, and red cell lifespan in 43 patients with myelodysplastic syndrome were studied by cluster analysis (Cazzola *et al.*, 1982) and the data resolved into three clusters. The poorest survival was found in the cluster with the lowest erythroid production, and the best survival in the cluster with erythroid hyperplasia and a high level of ineffective erythropoiesis. More recent studies showed ineffective erythropoiesis to be an early manifestation of MDS (May *et al.*, 1985) and erythroid hyperplasia seen in initial marrow aspirates to be associated with the longest survival (Jacobs & Clark, 1987).

### *Cytogenetic abnormalities*

Karyotypic abnormalities in haemopoietic cells are common, being found in about 50% of cases by most workers (Sokal *et al.*, 1980; Jacobs *et al.*, 1986). A higher incidence has been found in some series (Yunis *et al.*, 1986), possibly due to differences in patient selection and in laboratory techniques. Many of the non-random chromosomal aberrations are similar to those found in some cases of acute myeloblastic leukaemia (AML), though certain abnormalities typical of specific subgroups of AML are rarely seen in MDS. These include the 8:21 translocation often found with acute myeloblastic leukaemia of FAB type M2, the 15:17 translocation often found with acute promyelocytic leukaemia (FAB type M3), and the 19:22 translocation of chronic granulocytic leukaemia.

The most common abnormalities are monosomy 5 or 5q–, monosomy 7 or 7q– and trisomy 8. Many others have been described. Patients with a normal karyotype usually have a longer survival (Todd & Pierre, 1986), those with an initial abnormality at the time of diagnosis tend to have a less stable karyotype, evolve into a 'more malignant' clinical group and have a poorer survival. Only a small minority of patients with erythroid hyperplasia and sideroblastic precursors have an abnormal keryotype, but those showing abnormalities usually have a rapid clinical evolution (Yunis *et al.*, 1986). The presence of monosomy 7 or 7 deletions, usually of the long arm, is associated with a rapidly progressive course and a poor survival (Yunis *et al.*, 1986). Patients with marrow morphology showing myeloid hyperplasia and an increased number of blast cells usually have a high incidence of karyotype abnormalities, often with multiple complex derangements. They have a very poor prognosis. Multiple independent clones are commonly found in the preleukaemic state.

Although some patients with primary MDS have a history of exposure to chemical agents this does not appear to relate to a specific clinical picture or chromosome defect. Patients treated with chemotherapy or radiotherapy have a high incidence of secondary MDS and AML with a uniformly poor prognosis. This particularly malignant form of preleukaemia is associated with a clonal abnormality of chromosomes 5 and 7 in most cases. The critical regions appear to be 5q23–q32 and 7q32–33 or 7q34–35 (Le Beau *et al.*, 1986). Chromosomes 1, 4, 12, 14 and 18 appear to be involved significantly more often in therapy-induced MDS and AML than in the same conditions occuring *de novo* (Le Beau *et al.*, 1986).

### *DNA content of bone marrow cells*

It is often difficult to evaluate either the frequency or the extent of cytogenetic aberrations in patients with preleukaemia because chromosome spreads suitable for analysis may be few in number and poor in quality (Nowell, 1982). When ploidy is measured in marrow cells by DNA content with high resolution flow cytometry (Barlogie *et al.*, 1983), aneuploidy is found in about half the patients (Clark *et al.*, 1986), those with a low percentage of blasts tending to be hyperdiploid. A few patients with increased blast cell numbers show two separate $G_0/G_1$ peaks. Sideroblastic anaemia appears to be associated with hyperdiploidy, while hypodiploidy was found most commonly in patients with high numbers of marrow blasts (Clark *et al.*, 1986). Patients with hypodiploid marrow cells had a significantly shorter survival time than other patients and hypodiploidy appeared to be a better indicator of prognosis than the marrow blast count. These data suggest that there is a relationship between the loss of chromosomal material and progression towards a leukaemic phenotype.

### *Haemopoietic progenitors*

Impaired *in vitro* growth of CFU-GM colonies from marrow cells has been widely observed (Milner *et al.*, 1977; Greenberg & Mara, 1979; Verma *et al.*, 1979) and in some cases growth patterns have been related to prognosis (Verma *et al.*, 1979; Spitzer *et al.*, 1979). Erythroid colony growth is usually decreased in preleukaemic marrows (Milner *et al.*, 1977; Amato & Kahn, 1983; May *et al.*, 1985) but in PASA patients a combination of impaired or absent erythroid growth with normal or high CFU-GM growth is common (Ruutu *et al.*, 1984; May *et al.*, 1985).

### *Clinical progression*

The evolution of one clinical type of MDS into another with a poorer prognosis and the eventual emergence of AML in a substantial number of cases is commonly observed. Fohlmeister *et al.* (1985*b*) have suggested, on the basis of sequential observations in individual patients, that in many cases an initial marrow hypoplasia is followed by a phase of erythroid hyperplasia and ineffective erythropoiesis. This, in turn, is succeeded by a phase of myeloid hyperplasia with the later emergence of circumscribed nests of blast cells, suggesting new subclones arising within the pre-existing dysplastic clone. This scheme is compatible with the association of sideroblastic change, erythroid hyperplasia and hyperdiploidy with early stage disease and a relatively good survival. It must be rare for a patient to proceed from a 'myeloid' to an 'erythroid' phase.

### Possible models

The multistage nature of malignant transformation is well recognised (Knudson, 1973; Land *et al.*, 1983; Klein & Klein, 1985), though even in a specific tumour type the nature and sequence of these steps may vary and the malignant phenotype may be reached by different routes. The gradual evolution of a haemopoietic stem cell through the sequential stages from an initial genetic insult, the development of preleukaemia and progression to leukaemia is clearly in accordance with this general model (Jacobs, 1985). The initially damaged stem cell may well be undetectable by conventional methods of examination and many such cells, or even minor clonal populations, may fail to survive either due to a metabolic disadvantage or through destruction by the host immune defences. A further genetic change may be necessary before growth control mechanisms are altered sufficiently to give the new clone a proliferative advantage over normal haemopoietic cells. Such an advantage and the consequent clonal expansion may be related to increased sensitivity to growth factors, autocrine stimulation or inhibition of normal haemopoiesis. Clinical, morphological and cytogenetic data suggest that the progress of preleukaemia is marked by greatly varying rates of clonal expansion, or by clonal evolution with new populations having greater malignant potential (Tricot *et al.*, 1985; Tomonaga *et al.*, 1984). Except in specific instances, such as post-chemotherapy marrow damage, the nature of the external attack is undefined, though more than one agent can be involved. Similarly we have no clear information regarding the possibility of inherited genetic lesions predisposing to malignant change (de Vinuesa *et al.*, 1985; Krontiris *et al.*, 1986).

The genetic lesions giving rise to the preleukaemic process may be of two types. Firstly, each step may be related to a specific abnormality resulting in gene activation, amplification or deletion. At least three relevant groups of genes have been described, oncogenes (transforming genes), suppressor genes (anti-oncogenes) (Green & Wyke, 1985) and modulator genes (Klein & Klein, 1985). The recent burst of information linking oncogene proteins to specific functional roles in the regulation of cell proliferation (Burgess, 1985) and their place in multistep carcinogenesis has made it attractive to suppose that defects in the function of these genes play an important, if not an essential, role in the development of malignancy (Land *et al.*, 1983; Deuel & Huang, 1984; Klein & Klein, 1985; Bradshaw, 1986). The evidence in relation to human disease is, however, circumstantial and some doubts have been expressed as to whether oncogene abnormalities are sufficient or even necessary to cause cancer (Duesberg, 1985; Editorial, 1986). Secondly the evolution of leukaemia is associated with increasing chromosomal instability characterised grossly by aneuploidy, translocations, marker chromosomes and, in some cases, a progressive loss of chromosomal material. Knuutila *et al.* (1984) suggest that in MDS chromosome instability is associated with hypodiploid clones and aberrations in chromosome 7, and Clark *et al.* (1986) have shown that hypodiploidy is associated with poor survival. Increasing genomic instability appears to be an important feature in the progression of preleukaemia and may well provide a mechanism for the emergence of multiple abnormal clones, usually with chromosome loss. Most of these will not survive but some will have increased malignant potential.

### *Functional abnormalities in MDS*

Both leukaemia and myelodysplasia are characterised by gradual expansion of an undifferentiated self perpetuating stem cell population with poorly differentiated progeny. Knowledge of the mechanisms controlling the balance between stem cell renewal and maturation is imperfect and it is still not clear whether differentiation is precisely programmed at a genetic level or whether it is coupled to proliferative activity by humoral interactions (Sachs, 1982; Nicola & Metcalf, 1985).

In clinical terms the earliest manifestations of MDS commonly include evidence of ineffective erythropoiesis associated with cell cycle abnormalities and premitotic cell death. An analogous, though not so well characterised, process probably occurs in myeloid precursors. At this stage an accompanying marrow hyperplasia compensates for cell loss and maintains the supply of end cells to the peripheral blood, though these may be functionally defective. This suggests that the normal feedback mechanisms regulating the flow of mature blood cells from the marrow are still maintained. At a later stage the production of mature cells gradually fails, either due to a growth factor/receptor abnormality or to the gradual expansion of the undifferentiated cell population, seen clinically as an increase in marrow blast cells.

It is tempting to try and implicate abnormalities of those genes, such as *myc*, thought to be related to cell cycle control in the early phase of MDS and to speculate on the possible role of *src* in stem cell self renewal (Boettiger & Dester, 1986) or *fos* in differentiation (Muller, 1986), but at present we have no evidence for the involvement of these oncogenes in the preleukaemic process. It is simplistic to suggest that only a few specific genes are involved in the heterogenous picture of myelodysplasia but the accumulating evidence that chromosomes 5, 7 and 8 are the most common sites for karyotype abnormalities does draw attention to the possible role of oncogenes at these locations. In addition to *fms* (5q34), coding for the M-CSF receptor (Sherr *et al.*, 1985), *myc* (8q24), *mos* (8q22) and met (7q21–q31), the gene for GM-CSF is located at 5q21–q32 (Huebner *et al.*, 1985), the A-chain of PDGF is coded on chromosome 7 (Betsholtz *et al.*, 1986) and M-CSF on chromosome 5 close to GM-CSF (Rowley, personal communication). However, activated N-*ras* has also been identified in human myeloid leukaemia (Bos *et al.*, 1985; Hirai *et al.*, 1985) and abnormal expression of other oncogenes has been observed (Blick *et al.*, 1984).

### *What is the target cell?*

Evidence of the underlying clonality of haemopoiesis in MDS comes from an abundance of karyotype data and a few well studied cases with G6PD heterozygosity (Jacobs, 1985). There is, however, no clear evidence regarding the nature of the clonogenic cell. The patient studied by Prchal *et al.*, (1978) showed the same G6PD isoenzyme in myeloid and erythroid cells, platelets, macrophages, T and B cells, suggesting a disordered pluripotential stem cell. In the patient of Abkowitz *et al.* (1984) T cells were polyclonal (B cells were not studied), suggesting either that the clonogenic cell had restricted lineage potential or that a substantial number of long-lived normal T cells persisted in the circulation. Raskind *et al.* (1984) investigated a patient with both enzyme and karyotypic evidence of clonality and showed that although B cell lines produced by Epstein–Barr virus transformation contained only the single G6PD isoenzyme found in erythroid and myeloid cells, none of them contained the clonal karyotype abnormality found in the marrow. These workers hypothesised that an initial lesion may be found in the pluripotent stem cells and a second lesion producing chromosome abnormalities may occur in one of its progeny.

Similar evidence from the myeloid leukaemias suggests that the transformed cell may have restricted lineage potential. Fialkow (1985) found that in 5 elderly women with acute myeloblastic leukaemia (AML) the leukaemic stem cell was pluripotential for myeloid, erythroid, megakaryocytic and probably B-cell lines. However, in 10 younger AML patients the abnormal stem cell appeared to be restricted to the myeloid lineage. Lineage restriction to the monocytic pathway has also been described (Ferraris *et al.*, 1984). There are two possible explanations for lineage restriction in the abnormal clone. Either the target for the leukaemogenic insult is already a partially committed cell or the multipotential stem cell is damaged in a way that determines its subsequent maturation pathway. Three steps can be defined in the evolution of some cases of preleukaemia, an initial genetic change producing an abnormal clone with a growth advantage, a second change associated with the appearance of an abnormal karyotype, possibly in a stem cell with a more restricted differentiation potential, and thirdly the emergence of a leukaemic clone. If we accept that in some cases MDS may evolve clinically from an initially erythroid phenotype, through to a myeloid and then a more primitive phenotype, we need to consider whether erythroid progenitors are specifically damaged at an early stage of the disorder, with subsequent damage occurring at other points in the haemopoietic stem cell system or whether the entire process can be explained by a specific sequence of stem cell lesions determining the observed disease progression.

It is interesting that in the hierarchy of haemopoietic cells proposed by Johnson (1981) in which stem cell maturation involves progressive, sequential loss of differentiation potential, the final stage in the process results in a progenitor cell only capable of producing erythroid colonies, the penultimate stage being bipotential for erythroid and megakaryocyte colonies. The clinical sequence described by Fohlmeister *et al.* (1985*b*) almost parallels this maturation sequence and suggests that an early lesion might affect an erythroid committed progenitor cell. Juvonen *et al.* (1986) have suggested that both erythroid and megakaryocyte colony formation are abnormal at an early stage in the clinical development of MDS, often when CFU-GM formation remains normal, again suggesting the possibility of a lesion in a bipotential progenitor cell. If this were so then, in the light of Johnson's (1981) model, one might speculate that the probability of any particular stem cell maturation stage being a susceptible target for leukaemogenic insults may be related to intrinsic physiological differences in the developmental hierarchy such as population size, mitotic activity or self renewal capacity. We still have no way of determining the number of insults or the number of targets.

It would be difficult to provide a precise model to describe the human preleukaemic process, and it is likely that not all cases follow the same pathway. However, our present knowledge of the clinical syndromes is entirely consistent with current hypotheses of the multistage development of malignancy and oncogene function. In addition, and perhaps more importantly, clinical preleukaemia provides us with a definitive model of human leukaemogenesis that can be explored in detail. We now need to characterise the earliest abnormalities, their progression and the interrelations between genetic lesions and functional changes in preleukaemic haemopoiesis.

### References

ABKOWITZ, J.L., FIALKOW, P.J., NIEBRUGE, D.J., RASKIND, W.H. & ADAMSON, J.W. (1984). Pancytopenia as a clonal disorder of a multipotent haemopoietic stem cell. *J. Clin. Invest.*, **73**, 258.

AMATO, D. & KHAN, N.R. (1983). Erythroid burst formation in cultures of bone marrow and peripheral blood from patients with refractory anaemia. *Acta Haematologica* **70**, 1.

BARLOGIE, B., RABER, M.N. & SCHUMANN, J. (1983). Flow cytometry in clinical cancer research. *Cancer Res.*, **43**, 3982.

BAROSI, G., CAZZOLA, M., MORANDI, S., STEFANELLI, M. & PERUGINI, S. (1978). Estimation of ferrokinetic parameters by a mathematical model in patients with primary acquired sideroblastic anaemia. *Br. J. Haematol.*, **39**, 409.

BENNETT, J.M., CATOVSKY, D., DANIEL, M.T. & 4 others. (1982). Proposals for the classification of the myelodysplastic syndromes. *Br. J. Haematol.*, **51**, 189.

BETSHOLTZ, C., JOHNSSON, A., HELDIN, C.-H. & 5 others. (1986). cDNA sequence and chromosomal localization of human platelet-derived growth factor A-chain and its expression in tumour cell lines. *Nature*, **320**, 695.

BLICK, M., WESTIN, E., GUTTERMAN, J. & 5 others. (1984). Oncogene expression in human leukaemia. *Blood*, **64**, 1234.

BLOCK, M., JACOBSON, L.O. & BETHARD, W.F. (1953). Preleukaemic acute human leukaemia. *J.A.M.A.*, **152**, 1018.

BOEHME, W.M., PIIRA, T.A., KURNICK, J.E. & BETHLENFALVAY, N.C. (1978). Acquired hemoglobin H in refractory sideroblastic anemia. *Arch. Int. Med.*, **138**, 603.

BOETTIGER, D. & DEXTER, T.M. (1986). Altered stem cell (CFU-S) function following infection of hematopoietic cells with a virus carrying V-src. *Blood*, **67**, 398.

BOOGAERTS, M.A., NELISSEN, V., ROELANT, C. & GOOSSENS, W. (1983). Blood neutrophil function in primary myelodysplastic syndromes. *Br. J. Haematol.*, **55**, 217.

BOS, J.L., TOKSOZ, D., MARSHALL, C.J. & 5 others. (1985). Amino-acid substitutions at codon 13 of the N-ras oncogene in human acute myeloid leukaemia. *Nature*, **315**, 726.

BRADSHAW, T.K. (1986). Cell transformation: The role of oncogenes and growth factors. *Mutagenesis*, **1**, 91.

BURGESS, A. (1985). Growth factors and oncogenes. *Immunol. Today*, **6**, 107.

CARROLL, A.J., POON, M.-C. & ROBINSON, N.C. (1986). Sideroblastic anemia associated with thrombocytosis and a chromosome 3 abnormality. *Cancer Genet. Cytogenet.*, **22**, 183.

CAZZOLA, A.M., BAROSI, G., BERZUINI, C. & 4 others (1982). Quantitative evaluation of erythropoietic activity in dysmyelopoietic syndromes. *Br. J. Haematol.*, **50**, 55.

CETTO, G.L., VETTORE, L., DE MATTEIS, M.C., PIGA, A. & PERONA, G. (1982). Erythrocyte cation content, globin chain synthesis and glucose metabolism in dysmyelopoietic syndromes. *Acta Haematologica*, **68**, 124.

CLARK, R.E., HOY, T.G. & JACOBS, A. (1985). Granulocyte and monocyte surface membrane markers in the myelodysplastic syndromes. *J. Clin. Pathol.*, **38**, 301.

CLARK, R., PETERS, S., HOY, T., SMITH, S., WHITTAKER, K. & JACOBS, A. (1986). Prognostic significance of hypodiploid haemopoietic precursors in myelodysplastic syndromes. *New Engl. J. Med.*, **314**, 1472.

COIFFIER, B., ADELEINE, P. & VIALA, J.J. (1983). Dysmyelopoietic syndromes: A search for prognostic factors in 193 patients. *Cancer*, **52**, 83.

DE VINUESA, M.L., LARRIPA, I., DE PARGAMENT, M.M. & DE SALUM, S.B. (1985). Heterochromatic variants and their association with neoplasias. II. Preleukaemic states. *Cancer Genet. Cytogenet.*, **14**, 31.

DEUEL, T.S. & HUANG, J.S. (1984). Roles of growth factor activities in oncogenesis. *Blood*, **64**, 951.

DUESBERG, P.H. (1985). Activated proto-onc genes: Sufficient or necessary for cancer? *Science*, **228**, 669.

EDITORIAL (1986). Growth factors and malignancy. *Lancet* **ii**, 317.

FERRARIS, A.M., BROCCIA, G., MELONI, T., CANEPA, L., SESSAREGO, M. & GAETANI, G.F. (1984). Clonal origin of cells restricted to monocytic differentiation in acute nonlymphocytic leukaemia. *Blood*, **64**, 817.

FIALKOW, P.J. (1985). Clonal development, heterogeneity, and multistep pathogenesis of human leukaemias. In *Leukaemia: Recent Advances in Biology and Treatment*, Gale, R.P. & Golde, D.W. (eds) p. 491. Alan R. Liss: New York.

FOHLMEISTER, I., FISCHER, R., MODDER, B., RISTER, M. & SCHAEFER, H.-E. (1985*a*). Aplastic anaemia and the hypocellular myelodysplastic syndrome: Histomorphological, diagnostic and prognostic features. *J. Clin. Pathol.*, **38**, 1218.

FOHLMEISTER, I., FISCHER, R. & SCHAEFER, H.-E. (1985*b*). Preleukaemic myelodysplastic syndromes (MDS): Pathogenetical considerations based on retrospective clinicomorphological sequential studies. *Anticancer Res.*, **5**, 179.

FOUCAR, K., LANGDON, R.M., ARMITAGE, J.O., OLSON, D.B. & CARROLL, T.J. (1985). Myelodysplastic syndromes. A clinical and pathological analysis of 109 cases. *Cancer*, **56**, 553.

FRANCIS & HOFFBRAND (1985). The myelodysplastic syndromes and preleukaemia. In *Recent Advances in Haematology 4*, Hoffbrand, A.V. (ed). Churchill-Livingstone.

FRISCH, B. & BARTL, R. (1986). Bone marrow histology in myelodysplastic syndromes. (Suppl. 45). *Scand. J. Haematol.*, **45**, 21.

GREEN, A.R. & WYKE, J.A. (1985). Anti-oncogenes. A subset of regulatory genes involved in carcinogenesis? *Lancet*, **ii**, 475.

GREENBERG, P. & MARA, B. (1979). The preleukaemic syndrome. Correlation of *in vitro* parameters of granulopoiesis with clinical features. *Amer. J. Med.*, **66**, 951.

GREENBERG, P.L. (1983). The smouldering myeloid leukaemic states. *Blood*, **61**, 103.

HAST, R. & REIZENSTEIN, P. (1977). Studies on human preleukaemia. 1. Erythroblast and iron kinetics in a regenerative anaemia with hypercellular bone marrow. *Scand. J. Haematol.*, **19**, 347.

HIRAI, H., TANAKA, S., AZUMA, M. & 5 others. (1985). Transforming genes in human leukaemia cells. *Blood*, **66**, 1371.

HUEBNER, K., ISOBE, M., CROCE, C., GOLDE, D.W., KAUFMAN, S.E. & GASSON, J.C. (1985). The human gene encoding GM-CSF is at 5q21–q32, the chromosome region deleted in the 5q− anomaly. *Science*, **230**, 1282.

JACOBS, A. (1985). Myelodysplastic syndromes: Pathogenesis, functional abnormalities and clinical implications. *J. Clin. Pathol.*, **38**, 1201.

JACOBS, A. & CLARK, R.E. (1987). Pathogenesis and clinical variations in the myelodysplastic syndromes. *Clin. Haematol.* (in press).

JACOBS, R.H., CORNBLEET, M.A., VARDIMAN, J.W., LARSON, R.A., LE BEAU, M.M. & ROWLEY, J.D. (1986). Prognostic implications of morphology and karyotype in primary myelodysplastic syndromes. *Blood*, **67**, 1765.

JOHNSON, G.R. (1981). Is erythropoiesis an obligatory step in the commitment of multipotential haemopoietic stem cells? In *Experimental Haematology Today*, Baum, S.J. *et al.* (eds) p. 13. Karger: Basel.

JUVONEN, E., PARTANEN, S., KNUUTILA, S. & RUUTU, T. (1986). Megakaryocyte colony formation by bone marrow progenitors in myelodysplastic syndromes. *Br. J. Haematol.*, **63**, 331.

KARSDORF, A., DRESCH, C., METRAL, J. & NAJEAN, Y. (1983). Prognostic value of the combined suicide level of granulocyte progenitors in the labelling index of precursors in preleukaemic state and oligoblastic leukaemias. *Leukaemia Res.*, **7**, 279.

KLEIN, G. & KLEIN, E. (1985). Evolution of tumours and the impact of molecular oncology. *Nature*, **315**, 190.

KNUDSON, A.G. (1984). Genetic predisposition to cancer. *Cancer Detect. Prevent.*, **7**, 1.

KNUUTILA, S., TEERENHOVF, L. & BORGSTORM, D.H. (1984). Chromosome instability is associated with hypodiploid clones in myelodysplastic syndromes. *Hereditas*, **101**, 19.

KRONTIRIS, T.G., DIMARTINO, N.A., COLB, M., MITCHESON, H.D. & PARKINSON, D.R. (1986). Human restriction length polymorphisms and cancer risk assessment. *J. Cell. Biochem.*, **30**, 319.

LAND, H., PARADA, L.F. & WEINBERG, R.A. (1983). Cellular oncogenes and multistep carcinogenesis. *Science*, **222**, 771.

LE BEAU, M.M., WESTBROOK, C.A., DIAZ, M.O., & 5 others. (1986). Evidence for the involvement of GM-CSF and fms in the deleation (5q) in myeloid disorders. *Science*, **231**, 984.

LEVINE, M.N., KUHNS, W.K., BOLK, T.A., BEYER, T.A. & ROSSE, W.F. (1984). Acquired alteration in the expression of blood groups in a patient with sideroblastic anaemia and chronic renal failure. *Transfusion*, **24**, 8.

LINTULA, R., RASI, V., IKKALA, E., BORGSTROM, G.H. & VUOPIO, P. (1981). Platelet function in preleukaemia. *Scand. J. Haematol.*, **96**, 65.

MALDONADO, J.E. & PIERRE, R.V. (1975). The platelets in preleukaemia and myelomonocytic leukaemia: Ultrastructural cytochemistry and cytogenetics. *Mayo Clinic Proc.*, **50**, 573.

MAY, S.J., SMITH, S.A., JACOBS, A., WILLIAMS, A. & BAILEY-WOOD, R. (1985). The myelodysplastic syndrome: Analysis of laboratory characteristics in relation to the FAB classification. *Br. J. Haematol.*, **59**, 311.

MILNER, G.R., TESTA, N.G., GEARY, C.G. & 4 others. (1977). Bone marrow culture studies in refractory cytopaenia and 'smouldering laukaemia'. *Br. J. Haematol.*, **35**, 251.

MITROU, P.S. & FISCHER, M. (1979). Cell proliferation in refractory anaemia with hyperplastic bone marrow (preleukaemia). In *Preleukaemia*, Schmalzl, F. & Hellreigel, K.-P. (eds) p. 78–90. Springer: Berlin.

MONTECUCCO, C., RICCARDI, A., TRAVERSI, E. & 4 others. (1983). Flow cytometric DNA content in myelodysplastic syndrome. *Cytometry*, **4**, 238.

MULLER, R. (1986). Proto-oncogenes and differentiation. *TIBS*, **11**, 129.

NICOLA, N.A. & METCALF, D. (1985). The colony stimulating factors and myeloid laukaemia. *Cancer Surveys*, **4**, 789.

NOWELL, P.C. (1982). Cytogenetics of preleukaemia. *Cancer Genet. Cytogenet.*, **5**, 265.

PETERS, S.W., CLARK, R.E., HOY, T.G. & JACOBS, A. (1986). DNA content and cell cycle analysis of bone marrow cells in myelodysplastic syndromes (MDS). *Br. J. Haematol.*, **62**, 239.

PRCHAL, J.T., THROCKMORTON, D.W., CARROLL, A.J., FUSON, E.W., GAMS, R.A. & PRCHAL, J.S. (1978). A common progenitor for human myeloid and lymphoid cells. *Nature*, **274**, 590.

RASKIND, W.H., TIRUMALI, N., JACOBSON, R., SINGER, J. & FAILKOW, P.J. (1984). Evidence for a multistep pathogenesis of a myelodysplastic syndrome. *Blood*, **63**, 1318.

RICARD, M.F., SIGAUX, F., IMBERT, M. & SULTAN, C. (1979). Complementary investigations in myelodysplastic syndromes. In *Preleukaemia*, Schmalzl, F. & Hellriegel, K.-P. (eds). Springer: Berlin.

RUUTU, T., PARTANEN, S., LINTULA, R., TEERENHOVI, L. & KNUUTILA, S. (1984). Erythroid and granulocyte-macrophage colony formation in myelodysplastic syndromes. *Scand. J. Haematol.*, **32**, 395.

SACHS, L. (1982). Control of growth and normal differentiation in leukaemic cells. Regulation of the developmental programme and restoration of the normal phenotype in myeloid laukaemia. *J. Cell Physiol.*, Suppl. **1**, 151.

SCHOFIELD, K.P., STONE, P.C.W., KELSEY, P., LEYLAND, M.J. & STUART, J. (1983). Quantitative cytochemistry of blood neutrophils in myelodysplastic syndromes and chronic granulocytic leukaemia. *Cell Biochem. Function*, **1**, 92.

SCOTT, C.S., CAHILL, A., BYNOE, A.G., AINLEY, M.J., HOUGH, D. & ROBERTS, B.E. (1983). Esterase cytochemistry in primary myelodysplastic syndromes and megaloblastic anaemias: Demonstration of abnormal staining patterns associated with dysmyelopoiesis. *Br. J. Haematol.*, **55**, 411.

SEIGNEURIN, D. & HOLLARD, D. (1981). Use of the tritiated thimidine-labelling index of the myeloblast-promyelocyte pool for the identification of the leukaemic population of oligoblastic leukaemia. *Acta Haematologica*, **66**, 181.

SHERR, C.J., RETTENMEIER, C.W., SACCA, R., ROUSSEL, M.F., LOOK, A.T. & STANLEY, E.R. (1985). The c-fms proto-oncogene product is related to the receptor for the mononuclear phagocyte growth factor, CSF-1. *Cell*, **41**, 665.

SOKAL, G., MICHAUX, J.L. & VAN DEN BEGHE, H. (1980). The karyotype in refractory anaemia and preleukaemia. *Clin. Haematol.*, **9**, 129.

SPITZER, G., VERMA, D.S., DICKE, K.A., SMITH, T. & McCREDIE, K.B. (1979). Subgroups of oligoleukaemia as identified by *in vitro* agar culture. *Leukaemia Res.*, **3**, 29.

TODD, W.M. & PIERRE, R.V. (1986). Preleukaemia: A long-term prospective study of 326 patients. (suppl. 45). *Scand. J. Haematol.*, **36**, 114.

TOMONAGA, M., TOMONAGA, Y., KUSANO, M. & ICHIMARU, U. (1984). Sequential kayotypic evolutions and bone marrow aplasia preceding acute myelomonocytic transformation from myelodysplastic syndrome. *Br. J. Haematol.*, **58**, 53.

TRICOT, G., BOOGAERTS, M.A., DE WOLF-PEETERS, C., VAN DEN BERGHE, H. & VERWHILGEN, R.L. (1985). The myelodysplastic syndromes: Different evolution patterns based on sequential morphological and cytogenetic investigations. *Br. J. Haematol.*, **59**, 659.

VALENTINE, W.N., KONRAD, P.N. & PAGLIA, D.E. (1973). Dyserythropoiesis, refractory anaemia and 'preleukaemia'. *Blood*, **59**, 857.

VERMA, D.S., SPITZER, G., DICKE, K.A. & McCREDIE, K.B. (1979). *In vitro* agar culture patterns in preleukaemia and their clinical significance. *Leukaemia Res.*, **3**, 41.

WICKRAMASINGHE, S.N. (1975). *Human Bone Marrow*, Blackwells: Oxford.

YUNIS, J.J., RYDELL, R.E., OKEN, M.M., ARNESEN, M.A., MAYER, M.G. & LOBELL, M. (1986). Refined chromosome analysis as an independent prognostic indicator in De Novo myelodysplastic syndromes. *Blood*, **67**, 1721.


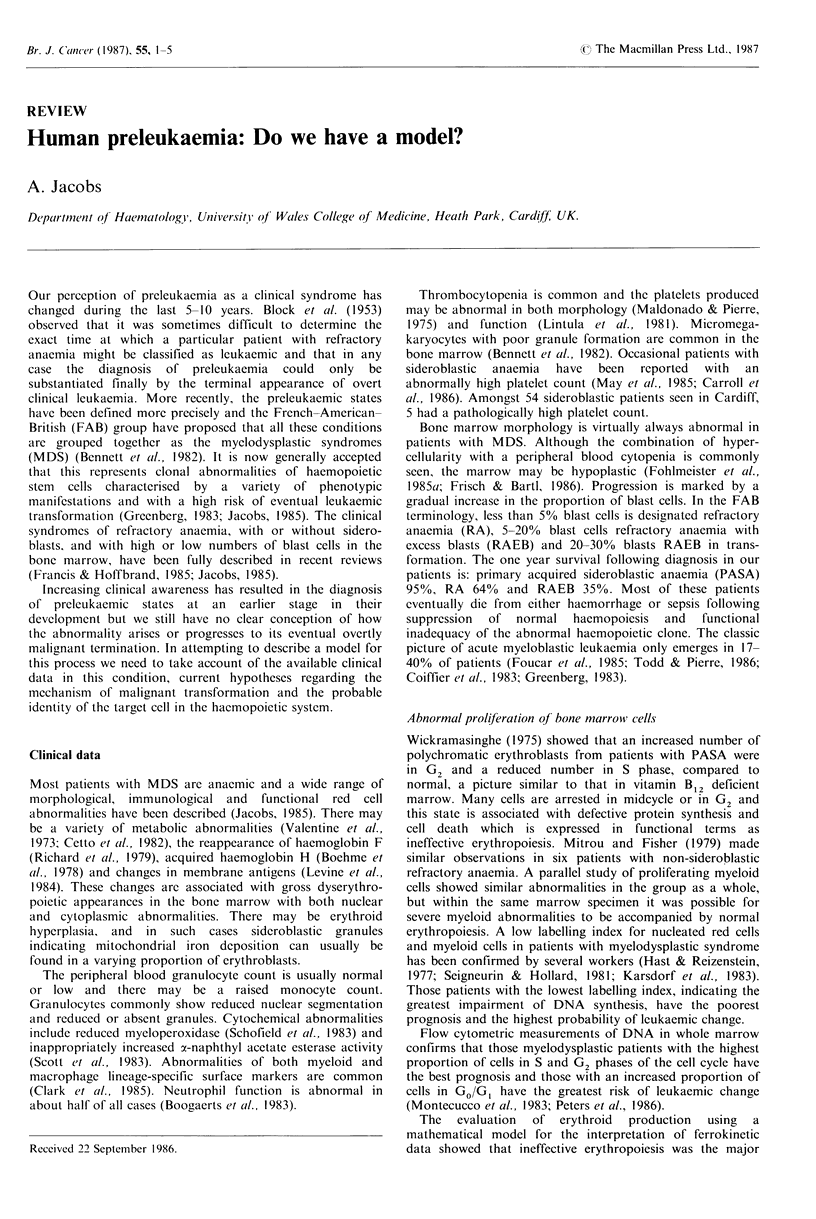

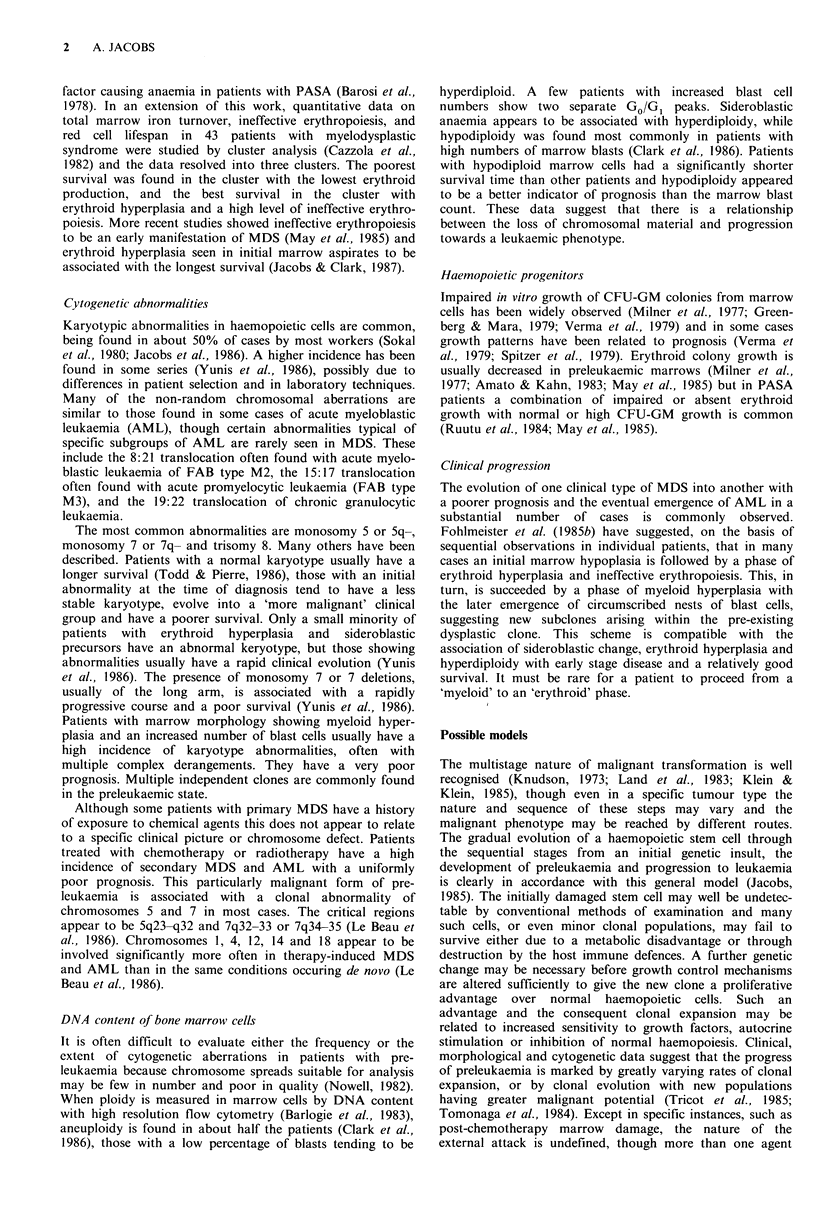

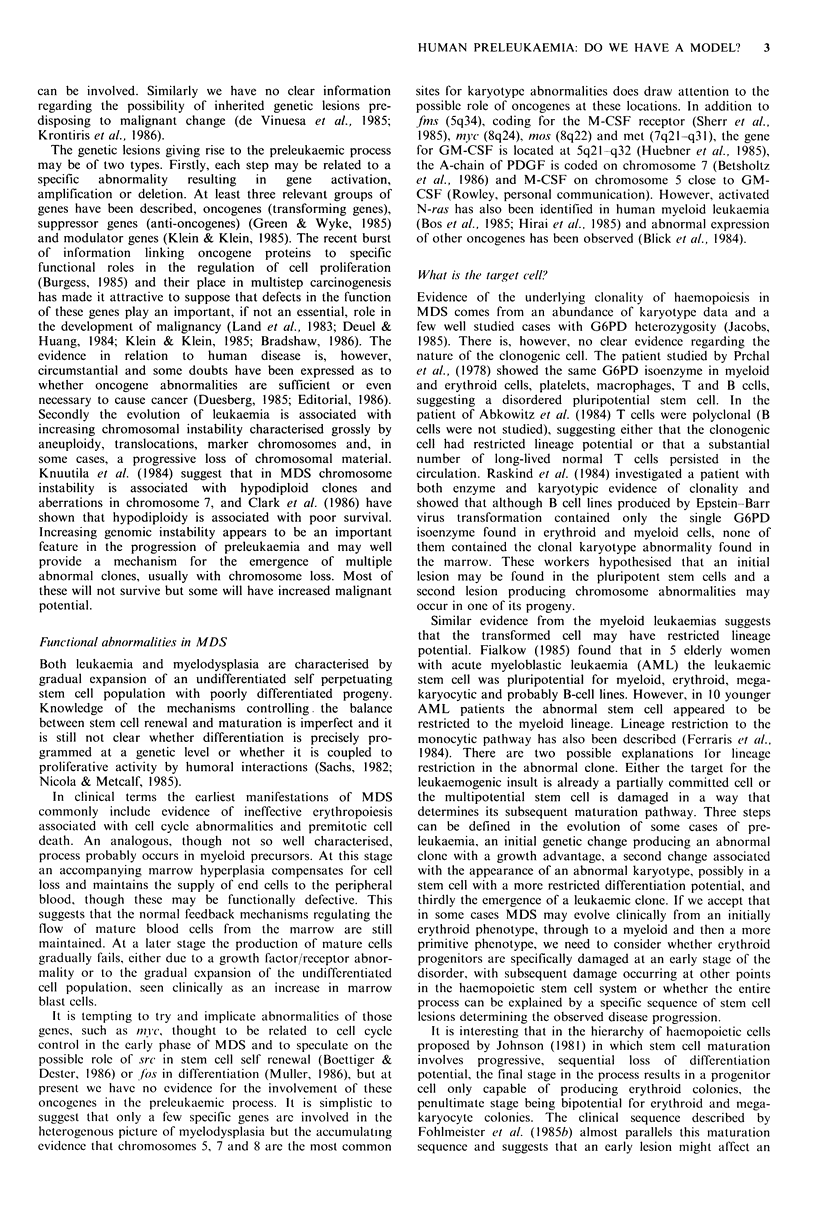

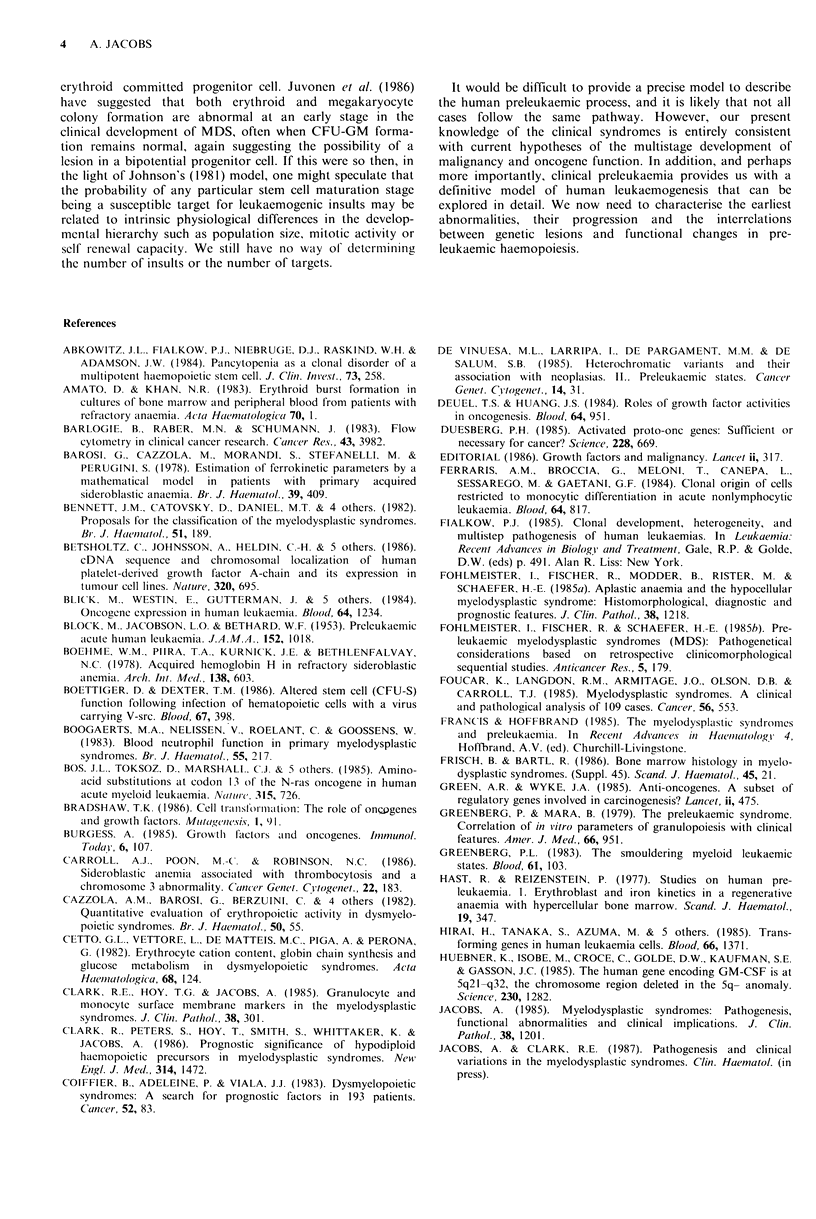

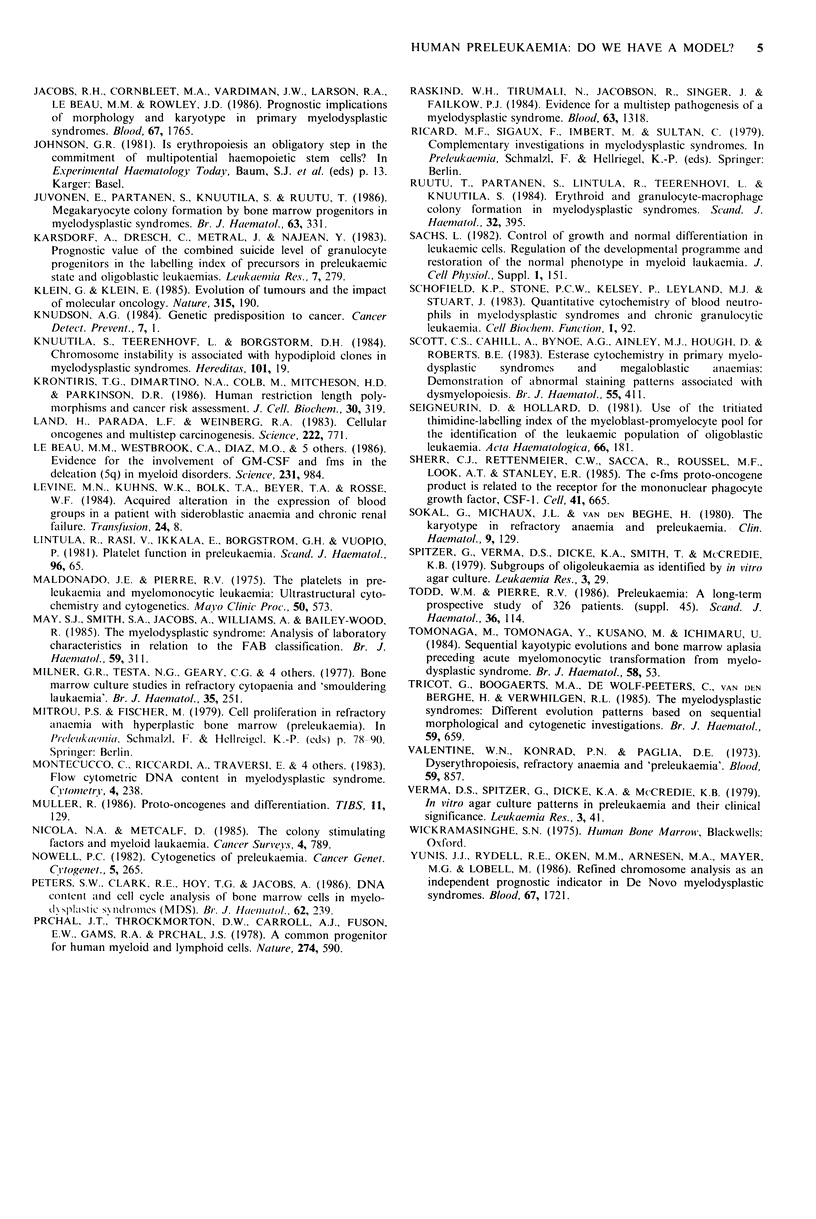


## References

[OCR_00478] Abkowitz J. L., Fialkow P. J., Niebrugge D. J., Raskind W. H., Adamson J. W. (1984). Pancytopenia as a clonal disorder of a multipotent hematopoietic stem cell.. J Clin Invest.

[OCR_00483] Amato D., Khan N. R. (1983). Erythroid burst formation in cultures of bone marrow and peripheral blood from patients with refractory anemia.. Acta Haematol.

[OCR_00513] BLOCK M., JACOBSON L. O., BETHARD W. F. (1953). Preleukemic acute human leukemia.. J Am Med Assoc.

[OCR_00488] Barlogie B., Raber M. N., Schumann J., Johnson T. S., Drewinko B., Swartzendruber D. E., Göhde W., Andreeff M., Freireich E. J. (1983). Flow cytometry in clinical cancer research.. Cancer Res.

[OCR_00492] Barosi G., Cazzola M., Morandi S., Stefanelli M., Perugini S. (1978). Estimation of ferrokinetic parameters by a mathematical model in patients with primary acquired sideroblastic anaemia.. Br J Haematol.

[OCR_00498] Bennett J. M., Catovsky D., Daniel M. T., Flandrin G., Galton D. A., Gralnick H. R., Sultan C. (1982). Proposals for the classification of the myelodysplastic syndromes.. Br J Haematol.

[OCR_00503] Betsholtz C., Johnsson A., Heldin C. H., Westermark B., Lind P., Urdea M. S., Eddy R., Shows T. B., Philpott K., Mellor A. L. (1986). cDNA sequence and chromosomal localization of human platelet-derived growth factor A-chain and its expression in tumour cell lines.. Nature.

[OCR_00509] Blick M., Westin E., Gutterman J., Wong-Staal F., Gallo R., McCredie K., Keating M., Murphy E. (1984). Oncogene expression in human leukemia.. Blood.

[OCR_00517] Boehme W. M., Piira T. A., Kurnick J. E., Bethlenfalvay N. C. (1978). Acquired hemoglobin H in refractor sideroblastic anemia. A preleukemic marker.. Arch Intern Med.

[OCR_00522] Boettiger D., Dexter T. M. (1986). Altered stem cell (CFU-S) function following infection of hematopoietic cells with a virus carrying V-src.. Blood.

[OCR_00527] Boogaerts M. A., Nelissen V., Roelant C., Goossens W. (1983). Blood neutrophil function in primary myelodysplastic syndromes.. Br J Haematol.

[OCR_00532] Bos J. L., Toksoz D., Marshall C. J., Verlaan-de Vries M., Veeneman G. H., van der Eb A. J., van Boom J. H., Janssen J. W., Steenvoorden A. C. Amino-acid substitutions at codon 13 of the N-ras oncogene in human acute myeloid leukaemia.. Nature.

[OCR_00537] Bradshaw T. K. (1986). Cell transformation: the role of oncogenes and growth factors.. Mutagenesis.

[OCR_00545] Carroll A. J., Poon M. C., Robinson N. C., Crist W. M. (1986). Sideroblastic anemia associated with thrombocytosis and a chromosome 3 abnormality.. Cancer Genet Cytogenet.

[OCR_00561] Clark R. E., Hoy T. G., Jacobs A. (1985). Granulocyte and monocyte surface membrane markers in the myelodysplastic syndromes.. J Clin Pathol.

[OCR_00566] Clark R., Peters S., Hoy T., Smith S., Whittaker K., Jacobs A. (1986). Prognostic importance of hypodiploid hemopoietic precursors in myelodysplastic syndromes.. N Engl J Med.

[OCR_00572] Coiffier B., Adeleine P., Viala J. J., Bryon P. A., Fière D., Gentilhomme O., Vuvan H. (1983). Dysmyelopoietic syndromes. A search for prognostic factors in 193 patients.. Cancer.

[OCR_00583] Deuel T. F., Huang J. S. (1984). Roles of growth factor activities in oncogenesis.. Blood.

[OCR_00587] Duesberg P. H. (1985). Activated proto-onc genes: sufficient or necessary for cancer?. Science.

[OCR_00593] Ferraris A. M., Broccia G., Meloni T., Canepa L., Sessarego M., Gaetani G. F. (1984). Clonal origin of cells restricted to monocytic differentiation in acute nonlymphocytic leukemia.. Blood.

[OCR_00605] Fohlmeister I., Fischer R., Mödder B., Rister M., Schaefer H. E. (1985). Aplastic anaemia and the hypocellular myelodysplastic syndrome: histomorphological, diagnostic, and prognostic features.. J Clin Pathol.

[OCR_00611] Fohlmeister I., Fischer R., Schaefer H. E. (1985). Preleukemic myelodysplastic syndromes (MDS): pathogenetical considerations based on retrospective clinicomorphological sequential studies.. Anticancer Res.

[OCR_00617] Foucar K., Langdon R. M., Armitage J. O., Olson D. B., Carroll T. J. (1985). Myelodysplastic syndromes. A clinical and pathologic analysis of 109 cases.. Cancer.

[OCR_00627] Frisch B., Bartl R. (1986). Bone marrow histology in myelodysplastic syndromes.. Scand J Haematol Suppl.

[OCR_00631] Green A. R., Wyke J. A. (1985). Anti-oncogenes. A subset of regulatory genes involved in carcinogenesis?. Lancet.

[OCR_00635] Greenberg P. L., Mara B. (1979). The preleukemic syndrome: correlation of in vitro parameters of granulopoiesis with clinical features.. Am J Med.

[OCR_00644] Hast R., Reizenstein P. (1977). Studies on human preleukaemia. I. Erythroblast and iron kinetics in aregenerative anaemia with hypercellular bone marrow.. Scand J Haematol.

[OCR_00650] Hirai H., Tanaka S., Azuma M., Anraku Y., Kobayashi Y., Fujisawa M., Okabe T., Urabe A., Takaku F. (1985). Transforming genes in human leukemia cells.. Blood.

[OCR_00654] Huebner K., Isobe M., Croce C. M., Golde D. W., Kaufman S. E., Gasson J. C. (1985). The human gene encoding GM-CSF is at 5q21-q32, the chromosome region deleted in the 5q- anomaly.. Science.

[OCR_00660] Jacobs A. (1985). Myelodysplastic syndromes: pathogenesis, functional abnormalities, and clinical implications.. J Clin Pathol.

[OCR_00672] Jacobs R. H., Cornbleet M. A., Vardiman J. W., Larson R. A., Le Beau M. M., Rowley J. D. (1986). Prognostic implications of morphology and karyotype in primary myelodysplastic syndromes.. Blood.

[OCR_00684] Juvonen E., Partanen S., Knuutila S., Ruutu T. (1986). Megakaryocyte colony formation by bone marrow progenitors in myelodysplastic syndromes.. Br J Haematol.

[OCR_00689] Karsdorf A., Dresch C., Metral J., Najean Y. (1983). Prognostic value of the combined suicide level of granulocyte progenitors and the labelling index of precursors in preleukemic states and oligoblastic leukemias.. Leuk Res.

[OCR_00695] Klein G., Klein E. (1985). Evolution of tumours and the impact of molecular oncology.. Nature.

[OCR_00699] Knudson A. G. (1984). Genetic predisposition to cancer.. Cancer Detect Prev.

[OCR_00703] Knuutila S., Teerenhovi L., Borgström G. H. (1984). Chromosome instability is associated with hypodiploid clones in myelodysplastic syndromes.. Hereditas.

[OCR_00708] Krontiris T. G., DiMartino N. A., Colb M., Mitcheson H. D., Parkinson D. R. (1986). Human restriction fragment length polymorphisms and cancer risk assessment.. J Cell Biochem.

[OCR_00577] Labal de Vinuesa M., Larripa I., Mudry de Pargament M., Brieux de Salum S. (1985). Heterochromatic variants and their association with neoplasias. II. Preleukemic states.. Cancer Genet Cytogenet.

[OCR_00712] Land H., Parada L. F., Weinberg R. A. (1983). Cellular oncogenes and multistep carcinogenesis.. Science.

[OCR_00716] Le Beau M. M., Westbrook C. A., Diaz M. O., Larson R. A., Rowley J. D., Gasson J. C., Golde D. W., Sherr C. J. (1986). Evidence for the involvement of GM-CSF and FMS in the deletion (5q) in myeloid disorders.. Science.

[OCR_00727] Lintula R., Rasi V., Ikkala E., Borgström G. H., Vuopio P. (1981). Platelet function in preleukaemia.. Scand J Haematol.

[OCR_00732] Maldonado J. E., Pierre R. V. (1975). The platelets in preleukemia and myelomonocytic leukemia. Ultrastructural cytochemistry and cytogenetics.. Mayo Clin Proc.

[OCR_00743] Milner G. R., Testa N. G., Geary C. G., Dexter T. M., Muldal S., MacIver J. E., Lajtha L. G. (1977). Bone marrow culture studies in refractory cytopenia and 'smouldering leukaemia'.. Br J Haematol.

[OCR_00754] Montecucco C., Riccardi A., Traversi E., Danova M., Ucci G., Mazzini G., Giordano P. (1983). Flow cytometric DNA content in myelodysplastic syndromes.. Cytometry.

[OCR_00763] Nicola N. A., Metcalf D. (1985). The colony-stimulating factors and myeloid leukaemia.. Cancer Surv.

[OCR_00767] Nowell P. C. (1982). Cytogenetics of preleukemia.. Cancer Genet Cytogenet.

[OCR_00771] Peters S. W., Clark R. E., Hoy T. G., Jacobs A. (1986). DNA content and cell cycle analysis of bone marrow cells in myelodysplastic syndromes (MDS).. Br J Haematol.

[OCR_00776] Prchal J. T., Throckmorton D. W., Carroll A. J., Fuson E. W., Gams R. A., Prchal J. F. (1978). A common progenitor for human myeloid and lymphoid cells.. Nature.

[OCR_00781] Raskind W. H., Tirumali N., Jacobson R., Singer J., Fialkow P. J. (1984). Evidence for a multistep pathogenesis of a myelodysplastic syndrome.. Blood.

[OCR_00792] Ruutu T., Partanen S., Lintula R., Teerenhovi L., Knuutila S. (1984). Erythroid and granulocyte-macrophage colony formation in myelodysplastic syndromes.. Scand J Haematol.

[OCR_00798] Sachs L. (1982). Control of growth and normal differentiation in leukemic cells: regulation of the developmental program and restoration of the normal phenotype in myeloid leukemia.. J Cell Physiol Suppl.

[OCR_00804] Schofield K. P., Stone P. C., Kelsey P., Leyland M. J., Stuart J. (1983). Quantitative cytochemistry of blood neutrophils in myelodysplastic syndromes and chronic granulocytic leukaemia.. Cell Biochem Funct.

[OCR_00810] Scott C. S., Cahill A., Bynoe A. G., Ainley M. J., Hough D., Roberts B. E. (1983). Esterase cytochemistry in primary myelodysplastic syndromes and megaloblastic anaemias: demonstration of abnormal staining patterns associated with dysmyelopoiesis.. Br J Haematol.

[OCR_00817] Seigneurin D., Hollard D. (1981). Use of the tritiated thymidine-labelling index of the myeloblast-promyelocyte pool for the identification of the leukemic population in oligoblastic leukemia.. Acta Haematol.

[OCR_00823] Sherr C. J., Rettenmier C. W., Sacca R., Roussel M. F., Look A. T., Stanley E. R. (1985). The c-fms proto-oncogene product is related to the receptor for the mononuclear phagocyte growth factor, CSF-1.. Cell.

[OCR_00829] Sokal G., Michaux J. L., van den Berghe H. (1980). The karyotype in refractory anaemia and pre-leukaemia.. Clin Haematol.

[OCR_00834] Spitzer G., Verma D. S., Dicke K. A., Smith T., McCredie K. B. (1979). Subgroups of oligoleukemia as identified by in vitro agar culture.. Leuk Res.

[OCR_00839] Todd W. M., Pierre R. V. (1986). Preleukaemia: a long-term prospective study of 326 patients.. Scand J Haematol Suppl.

[OCR_00844] Tomonaga M., Tomonaga Y., Kusano M., Ichimaru M. (1984). Sequential karyotypic evolutions and bone marrow aplasia preceding acute myelomonocytic transformation from myelodysplastic syndrome.. Br J Haematol.

[OCR_00850] Tricot G., Boogaerts M. A., De Wolf-Peeters C., Van den Berghe H., Verwilghen R. L. (1985). The myelodysplastic syndromes: different evolution patterns based on sequential morphological and cytogenetic investigations.. Br J Haematol.

[OCR_00857] Valentine W. N., Konrad P. N., Paglia D. E. (1973). Dyserythropoiesis, refractory anemia, and "preleukemia:" metabolic features of the erythrocytes.. Blood.

[OCR_00862] Verma D. S., Spitzer G., Dicke K. A., McCredie K. B. (1979). In vitro agar culture patterns in preleukemia and their clinical significance.. Leuk Res.

[OCR_00871] Yunis J. J., Rydell R. E., Oken M. M., Arnesen M. A., Mayer M. G., Lobell M. (1986). Refined chromosome analysis as an independent prognostic indicator in de novo myelodysplastic syndromes.. Blood.

